# Resveratrol improves alcoholic fatty liver disease by downregulating HIF-1α expression and mitochondrial ROS production

**DOI:** 10.1371/journal.pone.0183426

**Published:** 2017-08-17

**Authors:** Zhenhua Ma, Yangmin Zhang, Qingchun Li, Meng Xu, Jigang Bai, Shengli Wu

**Affiliations:** 1 Department of Hepatobiliary Surgery, the First Affiliated Hospital of Xi'an Jiaotong University, Xi'an, Shaanxi, P.R. China; 2 Department of Blood Transfusion, Xi’an Central Hospital, Xi'an, Shaanxi, P.R. China; 3 The Third Hepatic Disease Ward, The Affiliated Xi'an Eighth Hospital, Medical College of Xi'an Jiaotong University, Xi'an, Shaanxi, P.R. China; University of Louisville School of Medicine, UNITED STATES

## Abstract

Oxidative stress has been demonstrated to be involved in the etiology of alcoholic fatty liver disease (AFLD). Previous studies had demonstrated that resveratrol (RES) could reduce oxidative stress by different mechanisms. However, the effect of RES on alcohol-induced fatty liver remains unclear. In the present study, a total of 48 male SD rats were divided into three groups: Control, AFLD, and RES groups. Rats were administered with either nothing or 65% vol/vol alcohol (5 ml/kg/day in the first three days, and then 10 ml/kg/day in the following days) with or without RES supplementation (250 mg/kg/day) for 4 weeks. Blood and liver tissue samples were collected and subjected to biochemical assays, histological examination, Western blot, and mitochondrial radical oxygen species (ROS) assays. In RES group, significant decreases in serum ALT and AST concentrations, fat deposition, triglyceride (TG) content, HIF-1α protein expression as well as mitochondrial ROS production in liver were observed when compared with AFLD group (all *p* <0.05). These results indicated that RES could alleviate the liver injury induced by alcohol and prevent the progression of AFLD. Down regulation of HIF-1α protein expression and mitochondrial ROS production in liver might be, at least part of, the underlying mechanisms.

## Introduction

Alcohol abuse is a serious public health problem that is associated with many diseases, including alcoholic liver disease (ALD), the major cause of death from alcohol consumption [1]. Alcoholic fatty liver disease (AFLD) is the earliest stage of ALD that is characterized by TG accumulation in hepatocytes [2] and could further develop towards more severe lesions such as alcoholic steatohepatitis (ASH) and alcoholic cirrhosis (AC) [3]. The pathogenesis of ALFD involves oxidative stress and disruptions of lipid metabolism [4]. Recently, it has been demonstrated that the response to hypoxia plays an essential role in the development of ALFD [5] and this in turn has been linked to mitochondrial radical oxygen species (ROS) generation [6]. Currently, the most effective AFLD treatment is ethanol abstinence and there are still few effective pharmacological treatments for patients afflicted with this disease. Therefore, new therapies are urgently needed to prevent the progression of AFLD, especially for patients not stop drinking.

Resveratrol (trans-3,5,4'-trihydroxystilbene, RES), a natural polyphenol, was found in various plant species such as berries, peanuts, and especially grape skins [7]. Numerous studies have demonstrated its diverse pharmacological activities, such as antitumor effect [8], anti-inflammatory activity [9], antiviral activity [10], and antioxidative effect [11]. Previous studies had demonstrated that RES could reduce oxidative stress by different mechanisms including chelation of metal catalysts, activation of antioxidant enzymes, and inhibition of nicotinamide adenine dinucleotide phosphate (NADPH) oxidases [12]. Our previous works also showed that RES could reduce ROS production in activated platelets [13] and inhibit HIF-1α expression in rat model [14]. However, the effect of RES on alcohol-induced fatty liver remains unclear.

In light of these, the present study aimed to investigate the protective and mechanistic effects of RES on AFLD in a rat model. The findings will provide some clues that RES could be a potential agent for the treatment of AFLD.

## Materials and methods

### Reagents

Resveratrol was purchased from Xi’an Sino-Herb Bio-technology Company (Xi’an, China). Dimethyl sulfoxide (DMSO) and RPMI-1640 were purchased from Wuhan Boster Biological Technology, Ltd. (Wuhan, China). The RES was dissolved and sterilized in DMSO and then diluted in RPMI-1640 to 5mg/mL.

### Animals and treatment

Male Sprague-Dawley (SD) rats 9–10 weeks old weighing 190–210 g were purchased from the Animal Center of Xi’an Jiaotong University (Xi’an, China). All rats were allowed free access to water and standard laboratory chow. Care was provided in accordance with the “Guide for the care and use of laboratory animals” (NIH publication No. 85–23, revised in 1996). The study was approved by the Xi’an Jiaotong University Institutional Animal Care and Use Committee. We estimated that a total of 24 rats would be needed to detect a difference between groups, with a two-tailed one-way analysis of variance (α = 0.05 and β = 0.10), when the forecasting fatty liver score in controls, AFLD rats, and AFLD rats treated with RES were 0, 3 [15], and around 1.5 [16], respectively. Since half of the animals would be euthanized for measurement of liver HIF-1α and the other half for liver mitochondrial ROS, we doubled the number of animals to a total of 48 rats in our study. These rats were randomly divided into three experimental groups (16 rats in each group) as follows: Control group: no alcohol intake and no RES treatment and received an isocaloric beverage containing maltodextrin; AFLD group: rats were administered with 65% vol/vol alcohol for 4 weeks, by gavage 5 ml/kg/day in the first three days, and then 10 ml/kg/day in the following days as previously described [17]; and RES group: rats in this group received the same alcohol feeding regimens as AFLD group, and received RES at a concentration of 250 mg/kg/day intragastrically with an interval of 4 hours after alcohol administration for 4 weeks. Rats were euthanized at 4 weeks with an overdose of pentobarbital (100 mg/kg IV) followed by exsanguinations, and the liver tissues and blood samples were collected for the following experiments.

### Biochemical assays

Blood alcohol levels were measured using a NAD-ADH kit (Sigma, St. Louis, MO) according to the manufacturer’s instructions. Serum aspartate aminotransferase (AST) and alanine aminotransferase (ALT) concentrations were measured using Hitachi AU5400 automatic biochemical analyzer (Hitachi Corp., Japan) and Roche Diagnostics kit (Roche, USA) according to the instructions.

### Analysis of liver histology and Oil Red O staining

Paraformaldehyde-fixed liver sections (5 μm) were stained with hematoxylin-eosin. A semiquantitative histological evaluation [18] was carried out by two experienced pathologist blinded to the treatment groups to assess the extent of steatosis under light microscope independently (Olympus, Olympus LX70, Japan). The grading ranged 0–3 where 0 = less than 5% of the parenchyma was involved, 1 = 5 to 33%, 2 = 34 to 66%, 3 = more than 66%.

Frozen liver tissue was embedded into OCT compound (Sakura, Tokyo, Japan) and cut into 5 μm sections. Commercially available kits (Beyotime, Shanghai, China) were used to stain sections. Images of the sections were collected using a light microscope (Olympus, Olympus LX70, Japan). Image Pro Plus software (version 6.0; Media Cybernetics, Inc., Rockville, MD, USA) was used to analyze the integrated optical density (IOD) of the Oil Red O‑stained areas.

### Measurement of TG content in liver

TG content in liver was determined as previously described [19]. Briefly, 250 mg of liver sample was homogenized in 1.5 mL of methanol, and then added with 5.0 ml of MTBE and shaken at room temperature for 1 h. Subsequently, 1.25 mL of high purity water was added and mixed for 10 min, followed by centrifuging at 1,000 g for 10 min, and the upper organic layer was collected. The aqueous layer was re-extracted with MTBE/methanol/water mixture (10/3/2.5 v/v/v; 2 mL) and the combined organic layers were dried under nitrogen. The extracted dried lipids were dissolved in a mixture of triton X-114/methanol (2:1v/v, 60 μL) and analyzed for triglyceride (L-Type TGH) using commercially available kits (Wako Diagnostics, Richmond, VA, USA).

### Western blot analysis

Total cellular protein was extracted from homogenized liver tissue by the use of tissue protein extraction buffer (Pierce, Rockford, IL, USA) containing protease inhibitors (Protease Inhibitor Cocktail 100X, Pierce). After determining its concentration, protein samples were subjected to sodium dodecyl sulfate/polyacrylamide gel electrophoresis and transferred to a nitrocellulose membrane (ECL, Amersham, Buckinghamshire, UK). The membranes were blocked for 1 h and then incubated with primary antibodies (1:3000) overnight at 4°C prior to incubation with a horseradish peroxidase (HRP)-conjugated secondary antibody (1:6000) for 2 h at room temperature. An enhanced chemiluminescence detection kit (Amersham, Piscataway, NJ, USA) was used to detect the enzyme-conjugated antibody. The signal was captured with a UVP BioSpectrum500 imaging system (UVP, Upland, CA, USA). Protein expression was quantified by densitometry and normalized to β-actin expression. Both anti-HIF-1α and anti-β-actin antibodies were obtained from Santa Cruz Biotechnology, Inc. (Santa Cruz, CA, United States).

### Liver mitochondria isolation and measurement of mitochondrial ROS

Mitochondria were prepared by differential centrifugation of liver homogenates using ice-cold mitochondria isolation buffer containing 250 mM sucrose,1 mM EDTA, and 5 mM Tris-HCl, pH 7.5 [20]. Mitochondrial protein concentrations were determined spectrophotometrically using the bicinchoninic acid assay (ThermoScientific, Pittsburg, PA).

ROS production at complex 1, complex III, and reverse flow of electrons in isolated mitochondria was measured using an Amplex Red Hydrogen Peroxide/Peroxidase assay kits as previously described [21]. Measurement of ROS levels was performed on a microplate reader (Thermo Fisher Scientific Inc.) at 560 nm excitation and 590 nm emission. All experiments were performed at 37°C.

### Statistical analysis

Continuous data were presented as mean±SD. Statistical differences were calculated by Student's *t*-test using SPSS 16.0 statistical software (SPSS Inc., Chicago, IL, United States). A *p* value <0.05 was considered statistically significant.

## Results

### Blood alcohol concentration and markers of liver injury

There was no difference in blood alcohol concentration between the AFLD group and RES group (147±24 mg/dL *vs*. 143±18 mg/dL, *P* = 0.85). Serum ALT and AST concentrations in different groups are shown in Table 1. At 4 weeks post alcohol administration, serum ALT and AST levels were significantly higher in AFLD group than in Control group (all *P*<0.05). Coadministration with RES (250 mg/kg/day) showed a significant decrease in levels of serum ALT and AST than in AFLD group (all *P*<0.05).

**Table 1:** Serum ALT and AST levels in different groups (mean±SD).

Groups	n	ALT (U/L)	AST (U/L)
Control	16	38.75±12.62	40.25±11.17
AFLD	16	148.74±25.36*	139.05±21.45*
RES	16	64.33±17.59**	68.12±19.74**

AST, Aspartate aminotransferase; ALT, Alanine aminotransferase.

*p<0.05 *vs*. Control group

**p<0.05 *vs*. AFLD group.

### Analysis of liver histology and Oil Red O staining

**Fig 1:**
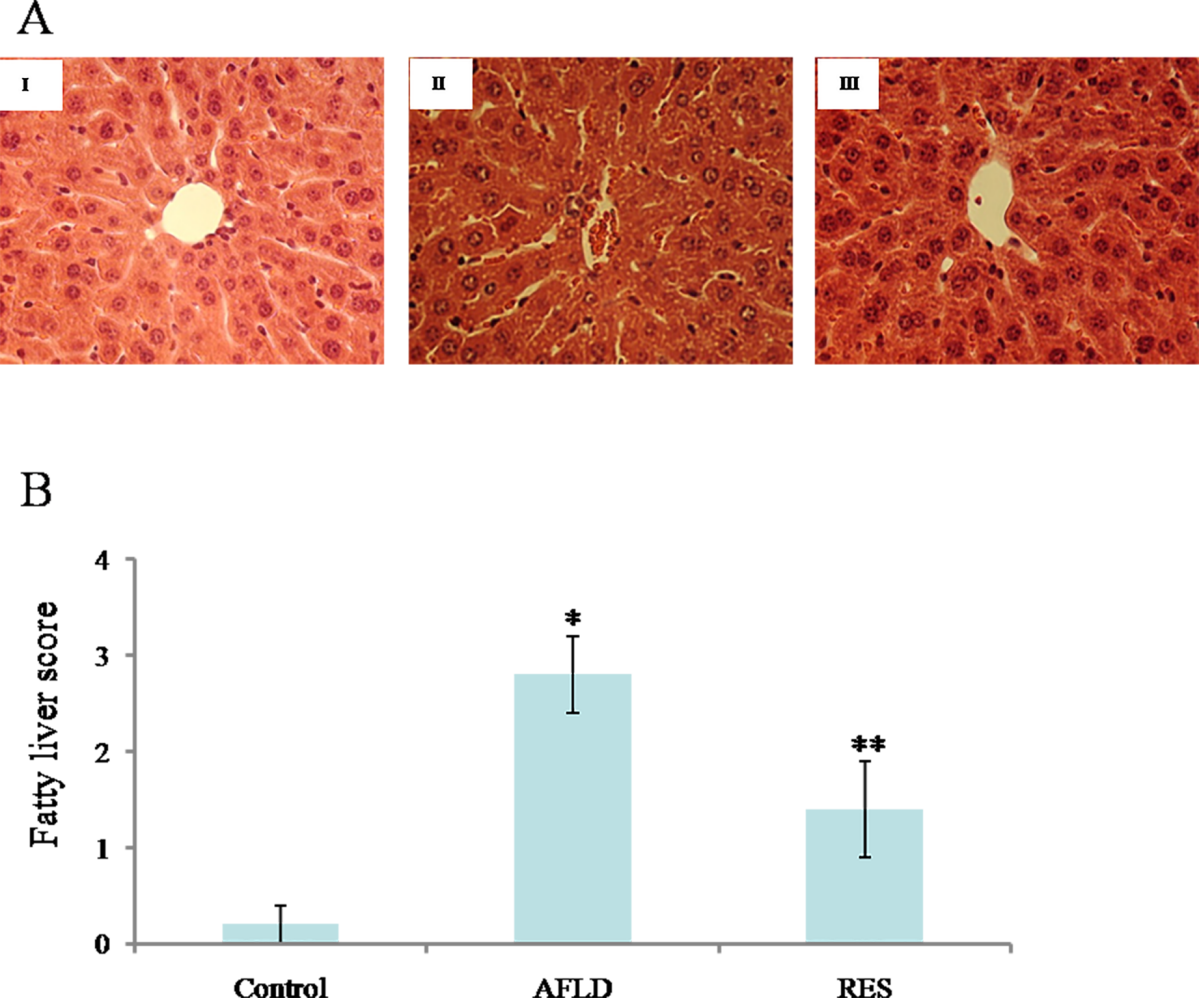
RES prevents hepatic macrosteatosis and microsteastosis in ethanol-fed animals. (A) Representative images of H&E-stained liver sections from Control (I), AFLD (II), and RES (III) groups. Images are at 400× magnification. (B) Fatty liver scores based on degree of lesions. Rats were administered with either nothing or 65% vol/vol alcohol (5 ml/kg/day in the first three days, and then 10 ml/kg/day in the following days) with or without RES supplementation (250 mg/kg/day) for 4 weeks. Values are represented as the mean ± SD (n = 16) (*p<0.05 *vs*. Control group; **p<0.05 *vs*. AFLD group.).

Chronic ethanol consumption increased hepatic macro- and microvesicular steatosis in AFLD group compared to Control group (Fig 1). Significant fat droplet accumulation was observed in the AFLD group [Fig 1A (II)], which resulted in a high fatty liver score (Fig 1B, *P*<0.05 *vs*. Control group). Supplementation with RES greatly decreased the number and size of fat droplet in ethanol-fed animals [Fig 1A (III)] and exhibited lower fatty liver scores compared to the AFLD group (Fig 1B, *P*<0.05).

**Fig 2:**
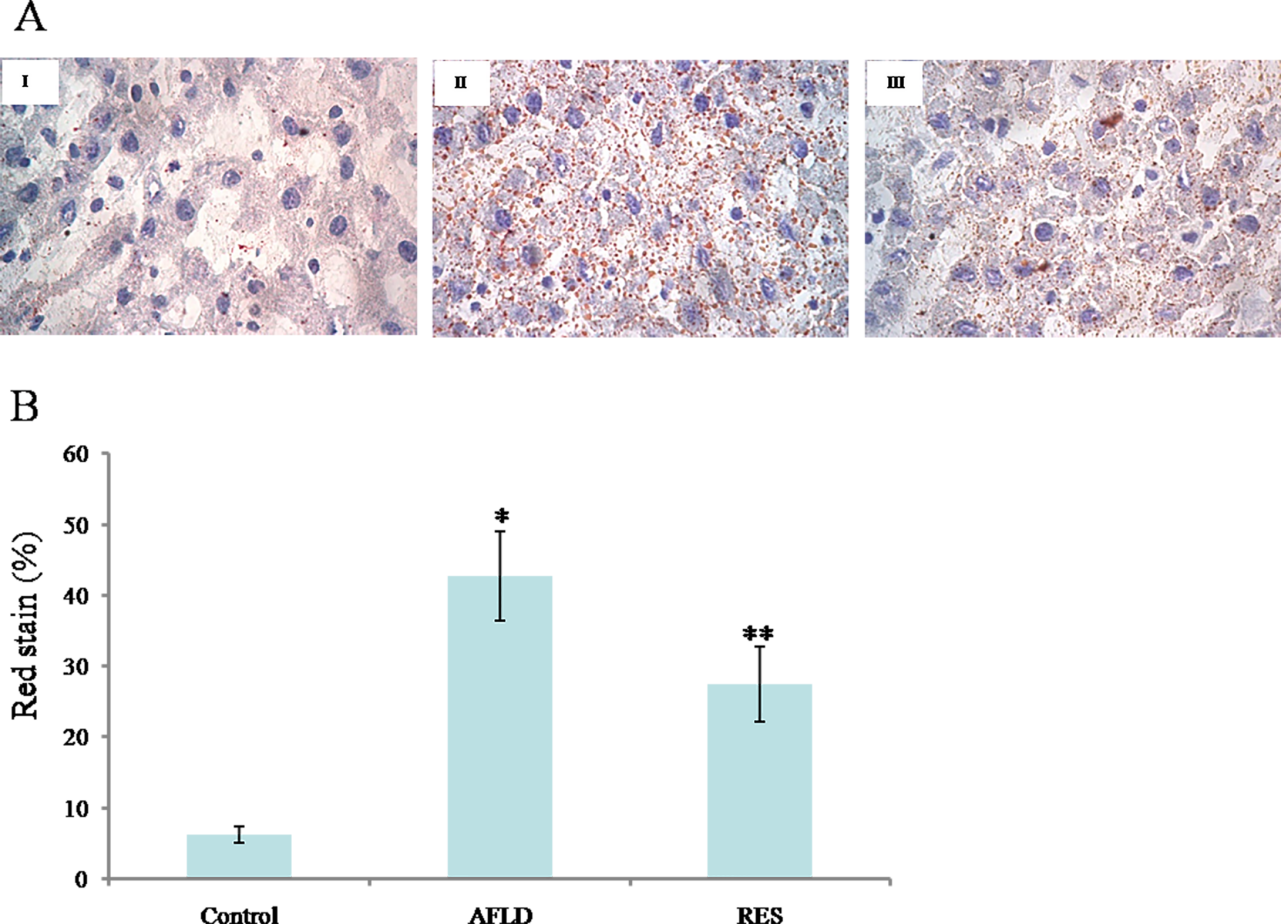
RES prevents lipid accumulation in hepatocytes in ethanol-fed animals. Representative images of Oil Red O-stained liver sections from Control (I), AFLD (II), and RES (III) groups. Images are at 400× magnification. (B) The IOD values of Oil Red O staining in the liver of rats. Rats were administered with either nothing or 65% vol/vol alcohol (5 ml/kg/day in the first three days, and then 10 ml/kg/day in the following days) with or without RES supplementation (250 mg/kg/day) for 4 weeks. Values are represented as the mean ± SD (n = 16) (*p<0.05 *vs*. Control group; **p<0.05 *vs*. AFLD group.).

Oil Red O staining of liver tissues demonstrated obvious intrahepatic lipid infiltration in AFLD rats, as indicated by an increased number of red hepatocytes and higher IOD values when compared with the Control rats (Fig 2). RES treatment significantly decreased lipid infiltration in AFLD rats (Fig 2A). In addition, the IOD values of the RES‑treated group were lower when compared with the AFLD group (Fig 2B).

### TG content in liver

**Fig 3:**
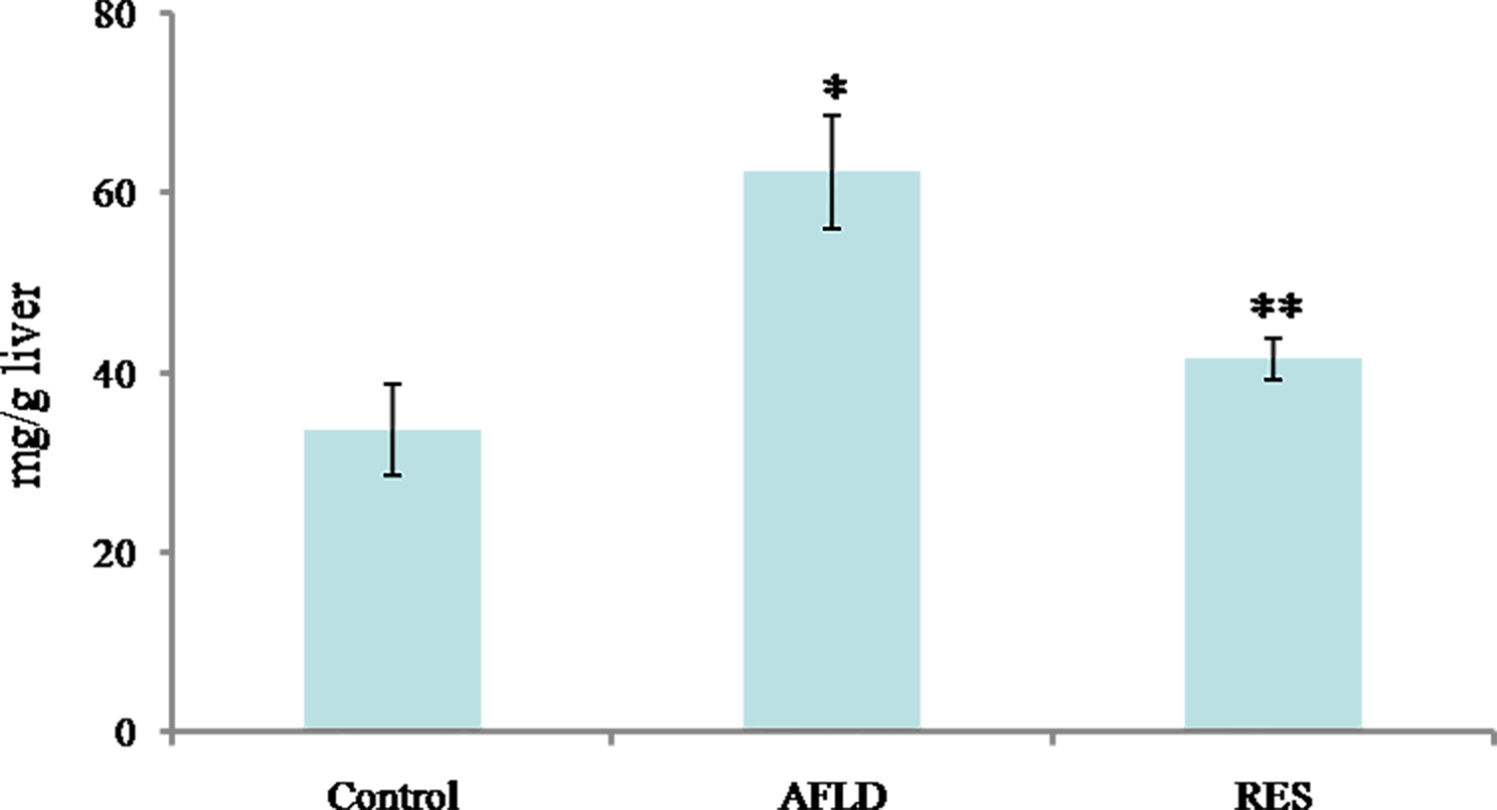
RES decreases TG accumulation in ethanol-fed rat livers. Biochemical analysis for TG accumulation in the indicated groups. Rats were administered with either nothing or 65% vol/vol alcohol (5 ml/kg/day in the first three days, and then 10 ml/kg/day in the following days) with or without RES supplementation (250 mg/kg/day) for 4 weeks. Values are represented as the mean ± SD (n = 16) (*p<0.05 *vs*. Control group; **p<0.05 *vs*. AFLD group.).

The TG content in rat livers was determined by biochemical analysis. Compared to Control group, TG accumulation was significantly increased in the livers of rats in AFLD group (*P*<0.05). Compared to AFLD group, a significant reduction in TG accumulation in RES group was observed (*P*<0.05; Fig 3).

### HIF-1α expression

**Fig 4:**
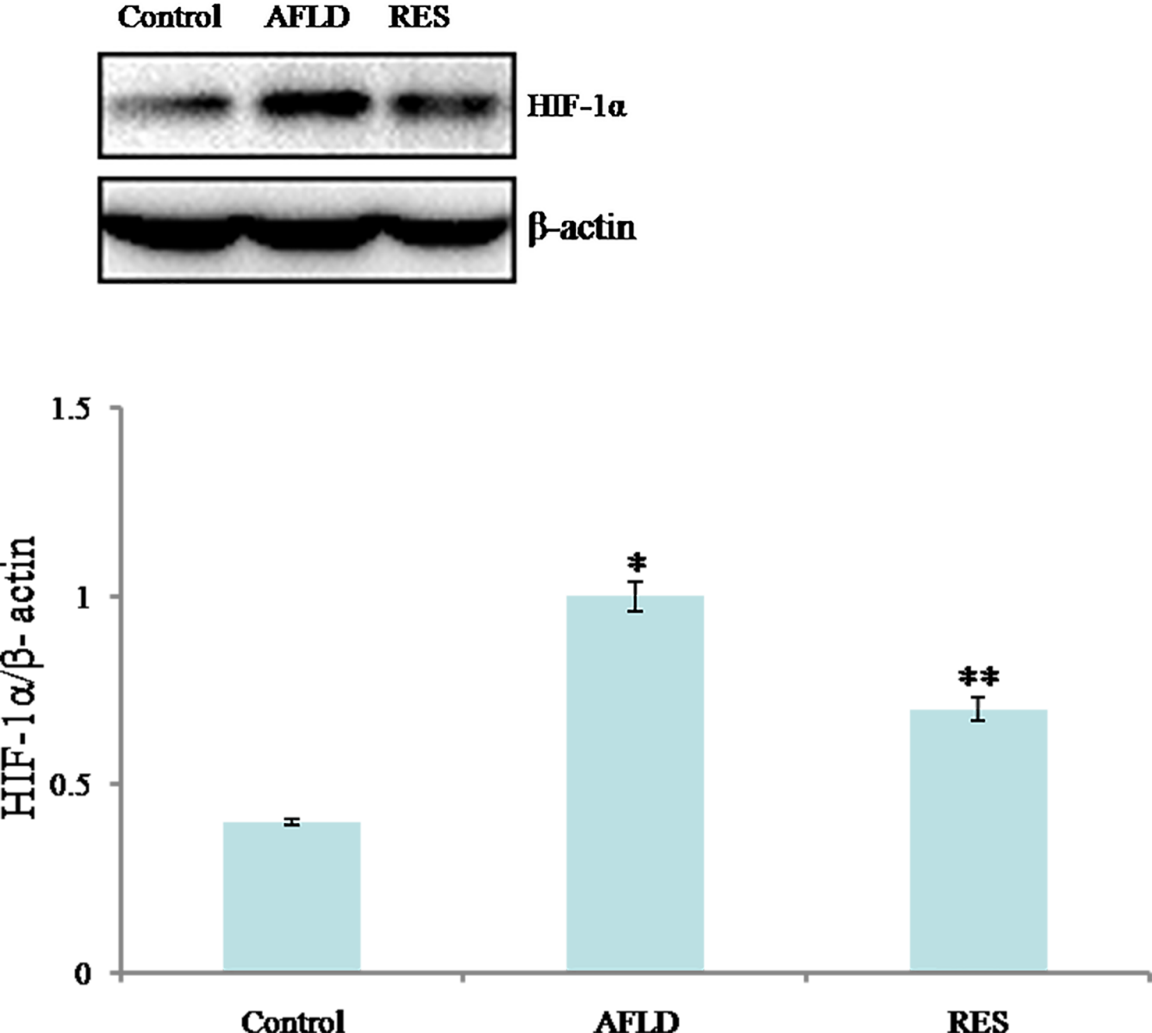
RES decreases the expression of HIF-1α protein in ethanol-fed rat livers. Western blot analysis for HIF-1α protein expression in the indicated groups. β-actin was used as a loading control. Rats were administered with either nothing or 65% vol/vol alcohol (5 ml/kg/day in the first three days, and then 10 ml/kg/day in the following days) with or without RES supplementation (250 mg/kg/day) for 4 weeks. Values are represented as the mean ± SD (n = 8) (*p<0.05 *vs*. Control group; **p<0.05 *vs*. AFLD group.).

The expression of HIF-1α in livers of experimental rats was examined by western blotting methods. Compared to Control group, the expression protein of HIF-1α was significantly increased in the livers of rats in AFLD group (*P*<0.05). Compared to AFLD group, a significant reduction in HIF-1α protein expression levels in RES group was observed (*P*<0.05; Fig 4).

### Production of mitochondrial ROS

**Fig 5:**
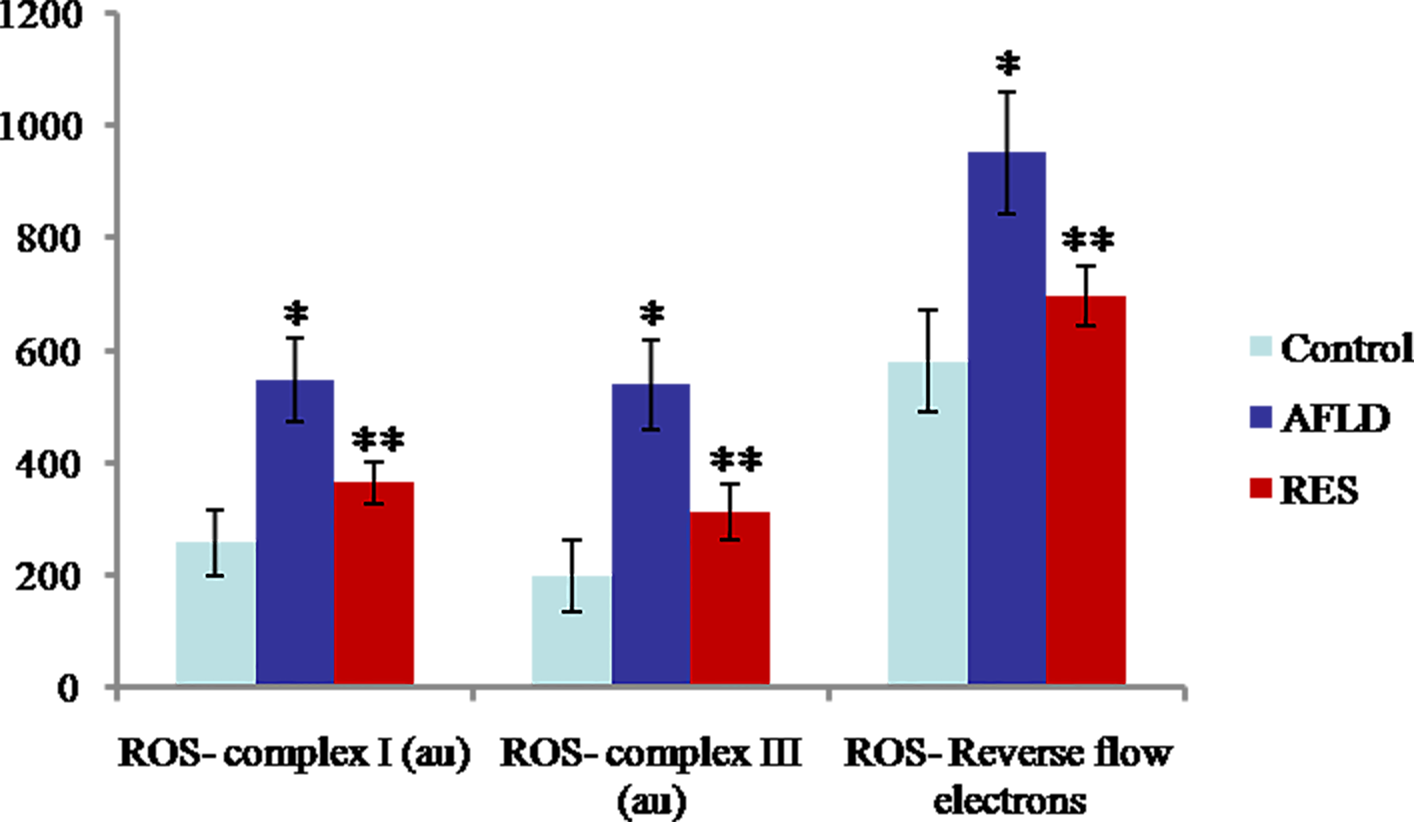
RES decreases mitochondrial ROS production in ethanol-fed rat livers. ROS production at complex 1, complex III, and reverse flow of electrons in isolated mitochondria in the indicated groups. Rats were administered with either nothing or 65% vol/vol alcohol (5 ml/kg/day in the first three days, and then 10 ml/kg/day in the following days) with or without RES supplementation (250 mg/kg/day) for 4 weeks. Values are represented as the mean ± SD (n = 8) (*p<0.05 *vs*. Control group; **p<0.05 *vs*. AFLD group.).

Compared to Control group, mitochondrial ROS production at complex I, complex III, and in reverse flow of electrons were all increased greatly in AFLD group (all *P*<0.05). Compared to AFLD group, a significant reduction in mitochondrial ROS production at complex I, complex III, and in reverse flow of electrons in RES group was observed (all *P*<0.05; Fig 5).

## Discussion

The etiology of AFLD is highly complex involving disruptions in multiple liver cell types, metabolic and signaling pathways, and sub-cellular organelle function [22]. Some recent studies had showed that chronic alcohol consumption depressed liver mitochondrial bioenergetics, increased mitochondrial ROS production and sensitivity of the mitochondrial permeability transition (MPT) pore in animal models [23–25]. A direct consequence of impaired bioenergetics is hepatocyte death, which will lead to dysregulated lipid clearance resulting in triglyceride accumulation. Under the physiological state, more than 90% of ROS are produced by the mitochondria [26]. Taken together, these finding revealed a main role of mitochondria in the development of AFLD and increased mitochondrial ROS production would occur as a consequence of alcohol-mediated alterations to the respiratory chain [27].

Studies have shown that these mitochondria-derived ROS are both necessary and sufficient to stabilize and activate hypoxia-inducible factor-1α (HIF-1α) [28]. HIF-1α was firstly described by Semenza in 1992 and its expression is tightly regulated by low oxygen tension [29]. Previous studies had demonstrated that induction of tissue hypoxia and HIF-1α in the liver is a hallmark of alcohol-induced liver disease [30]. The alcohol-induced chemokine MCP-1 triggers lipid accumulation in hepatocytes via HIF-1α activation, suggesting HIF-1α in hepatocytes is a major determinant in the pathogenesis of alcoholic steatosis [5].

RES is a phenolic compound which possesses a variety of biochemical and physiological effects including antioxidant, anti-aging, regulation of lipid metabolism, anti-cancer properties [31]. Previous studies had demonstrated that RES could act as a scavenger of hydroxyl, superoxide, and other radicals [32]. A recent study showed that RES could reduce HIF-1α accumulation and prevent fibrosis in hypoxic adipose tissue [33]. In our previous study, we also found that RES could reduce ROS production in activated platelets in a rat fibrosis model [13] and inhibit HIF-1α expression in liver tissue in a rat ischemia-reperfusion model [14]. In the present study, the protective effects of RES on alcohol-induced fatty liver were investigated in a rat model. As expected, our results showed that compared with AFLD group, significant decreases in serum ALT and AST concentrations and fat deposition were observed in RES group. Moreover, we further showed that the TG content and HIF-1α protein expression and mitochondrial ROS production in liver decreased significantly in RES group when compared to AFLD group. These findings affirmed the results of previous studies showing the hepatoprotective effects of RES. More importantly, we provided the first evidence supporting the protective effects of RES in the setting of alcohol-induced fatty liver disease, which are consistent with previous findings, and down regulation of HIF-1α protein expression and mitochondrial ROS production in liver might be, at least part of, the underlying mechanisms. However, additional studies are needed to identify the detailed mechanisms by which RES regulated the expressions of HIF-1α and mitochondrial ROS production in liver.

Taken together, our present study has provided evidence that RES, exerts its hepatoprotective effects through inhibiting HIF-1α and mitochondrial ROS production in liver in a rat model of AFLD. ROS/HIF-1a axis, as a key regulator of alcohol-induced fatty liver disease, could be a promising drug target for RES in the development of effective agent for the treatment of AFLD. Future studies are required to further investigate the molecular mechanisms underlying the involvement of ROS/HIF-1a axis in AFLD such as the effect of RES on pan-inhibitor of HIF prolyl hydroxylase.
